# CTCF as a boundary factor for cohesin-mediated loop extrusion: evidence for a multi-step mechanism

**DOI:** 10.1080/19491034.2020.1782024

**Published:** 2020-07-07

**Authors:** Anders S. Hansen

**Affiliations:** Department of Biological Engineering, Massachusetts Institute of Technology, Cambridge, MA, USA

**Keywords:** CTCF, Cohesin, TADs, Loop Extrusion, Convergent Rule, Binding Polarity, RNA-Binding Region, PDS5, NIPBL

## Abstract

Mammalian genome structure is closely linked to function. At the scale of kilobases to megabases, CTCF and cohesin organize the genome into chromatin loops. Mechanistically, cohesin is proposed to extrude chromatin loops bidirectionally until it encounters occupied CTCF DNA-binding sites. Curiously, loops form predominantly between CTCF binding sites in a convergent orientation. How CTCF interacts with and blocks cohesin extrusion in an orientation-specific manner has remained a mechanistic mystery. Here, we review recent papers that have shed light on these processes and suggest a multi-step interaction between CTCF and cohesin. This interaction may first involve a pausing step, where CTCF halts cohesin extrusion, followed by a stabilization step of the CTCF-cohesin complex, resulting in a chromatin loop. Finally, we discuss our own recent studies on an internal RNA-Binding Region (RBRi) in CTCF to elucidate its role in regulating CTCF clustering, target search mechanisms and chromatin loop formation and future challenges.

## Introduction

Mammalian genomes face the dual challenge of safely packaging and storing around two meters of DNA inside the nucleus, while retaining access to several processes including transcription, replication and DNA repair. Accordingly, genome structure is intimately linked to genome function and mammalian genomes are organized at multiple scales. At the chromosomal scale, chromosomes occupy discrete territories [1] and at the small scale, 147 base pairs of DNA are wrapped around histone octamers into nucleosomes [2]. In this review, we will focus on the intermediate scale of mammalian interphase genome organization (kilobases to megabases), where genomes appear to be organized by two major mechanisms [3].

**Figure 1.**
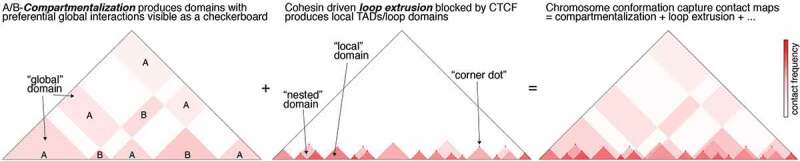
**A simplified illustration of how contact map features are shaped by A/B-compartmentalization and loop extrusion**. Highly simplified sketches of hypothetical contact maps produced by chromosome conformation capture methods such as Hi-C. Left: Hypothetical contact map produced by A/B-compartmentalization. Compartmentalization generates both local and global domains. Middle: Hypothetical contact map produced by loop extrusion. Loop extrusion generates strictly local maps demarcated by strong convergent CTCF binding sites, and sometimes forms nested domains. Right: Real contact maps are affected by both A/B-compartmentalization and CTCF/cohesin-mediated loop extrusion – as well as a number of other processes especially at the fine-scale [129,130] – and are therefore the sum of all of these processes. This makes interpreting and classifying ‘domains’ in Hi-C contact maps highly challenging [9,30].

First, a poorly understood ***compartmentalization*** mechanism organizes the genome into two major A- and B-compartments [4,5]. A-compartments largely correspond to gene-rich and transcriptionally active euchromatic regions, that replicate earlier in S-phase and tend to associate with nuclear speckles [6,7]. In contrast, B-compartments largely correspond to gene-poor condensed heterochromatin that is largely transcriptionally inactive, tends to replicate late in S-phase, and is often associated with the nuclear lamina and the nucleolus [8–10]. A/B compartments can be further divided into several subcompartments [11–14]. While the molecular mechanisms of compartmentalization remain poorly understood, preferential A-A and B-B interactions can largely explain the segregation of chromosomes into compartments. In fact, polymers of distinct A/B segments, known as block copolymers, naturally undergo microphase separation [3,12,15]. As such, compartmentalization leads to global segregation. Loci in an A compartment on a given chromosome are more likely to interact with other A compartment loci both on the same chromosome and on other chromosomes. By causing both preferential intra- and inter-chromosomal interactions, A/B compartmentalization is visible as a ‘plaid’ or ‘checkerboard’ pattern in Hi-C contact maps [4,5] (Figure 1).

Second, increasing evidence suggests that a ***loop extrusion*** mechanism organizes genomes into local domains known as Topologically Associating Domains (TADs) or Loop Domains [16–19]. During interphase, the multi subunit cohesin complex [20] is hypothesized to extrude DNA bi-directionally at a speed of ~0.5–1.0 kb/second [16–19,21–23], until it is blocked by a boundary. The insulator protein, CCCTC-Binding Factor (CTCF), appears to be the primary boundary factor in mammals, at least at the level of TADs and loops visible in Hi-C [24]. This model is supported by the observations that loss of cohesin [25–28] eliminates essentially all TADs and loop domains as measured by Hi-C, whereas loss of CTCF affects many TADs and loop domains [28,29], though the effect of CTCF loss on TADs is markedly weaker than cohesin loss. Unlike A/B-compartments, TADs are local domains, albeit often nested local domains (Figure 1). We note that nomenclature and domain classification remain a challenge in the field: although TADs appear to be formed by loop extrusion, not all TADs are anchored by visible loops (‘corner dots’) and not all loops give rise to TADs. Furthermore, since TADs and compartment domains can appear similar in contact maps, distinguishing these is also a challenge [9,30].

Notably, CTCF binding sites are asymmetric and CTCF-mediated loops are largely bridged by convergent CTCF DNA binding sites [11,31–33]. Moreover, inversion of a single CTCF binding site can be sufficient to disrupt a loop and TAD, and rearrange the 3D folding of hundreds of kilobases of DNA [18,31,32,34], though not all inversions had this effect [31,32]. What this means is that a loop-extruding cohesin complex with a diameter of ~50 nm [20] is somehow able to distinguish whether it approaches a comparatively tiny DNA-bound CTCF protein (~3-5 nm) from the N-terminal or C-terminal side [35]. Though how this works remains a mechanistic mystery, a series of very recent papers have shed new light on this process [28,36–42].

In this Extra View article, we will focus on how CTCF and cohesin interact to regulate genome folding into TADs and loops, and place our own recent studies in the context of these new findings [36,43]. For other important aspects of 3D genome organization including the role of TADs in regulating gene expression and other functions, how to interpret Hi-C contacts maps, nomenclature, distinguishing TAD and compartment domains (Figure 1), the wealth of available technologies for probing nuclear organization, and other exciting areas, we refer the reader to a number of excellent recent reviews [9,30,44–46].

### Cohesin

**Figure 2.**
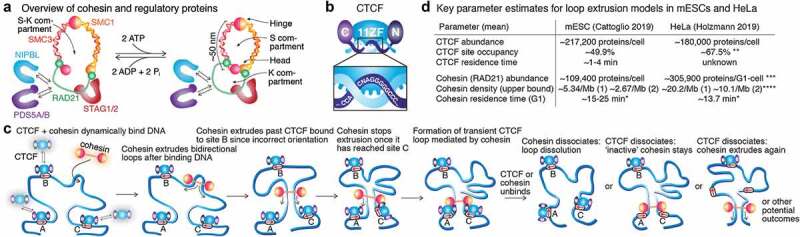
**Overview of cohesin, CTCF, and loop extrusion**. (a) Overview of mammalian cohesin and some of its regulatory proteins. (b) Overview of CTCF with N-terminal, 11 Zinc Fingers, and C-terminal domains. (c) Simplified sketch of cohesin-mediated loop extrusion and the convergent CTCF rule. (d) Summary of key parameters constraining loop extrusion models in mouse embryonic stem cells (mESCs) [65] and human HeLa cells [83], with mESC residence times taken from [70]. * These are cohesin G1 residence times (both STAG1 and STAG2), but after these studies were published it was found that STAG2-cohesin binds DNA substantially more dynamically than STAG1-cohesin [38], suggesting that putative loop extruding G1 cohesins have at least two residence times. ** Estimated from [83] (~180,000 and ~120,000 CTCF proteins and sites per HeLa cell) with added assumption that 45% of CTCF proteins are bound to cognate sites (~45%, i.e. mean of mESC and U2OS in [70]). *** 305,900 is the mean of the LC-MS and FCS estimates reported in [83]. **** Cohesin density is estimated from ~159,437 dynamically bound (~13.7 min residence time) cohesin proteins (SCC1-mEGFP) in G1 and the reported HeLa genome sizes 7.9 Gb, both taken from [83]. It is important to note that these are genomic averages: e.g. CTCF residence time is for an average site (some sites will have slower and faster binding), cohesin density may not be uniform throughout the genome, and since the two *in vitro* cohesin loop extrusion papers disagreed on whether cohesin is monomeric [23] or dimeric [21], densities for both monomeric [1] and dimeric [2] are shown.

Cohesin belongs to the Structural Maintenance of Chromosomes (SMC) family of protein complexes. SMC complexes appear to organize chromosomal DNA topology in all living organisms from bacteria to eukaryotes. SMC complexes are ancient and their evolution likely preceded histones [20]. Here, we will focus on mammalian cohesin, which contains two proteins, SMC1 and SMC3, that dimerize at the hinge (Figure 2a). Antiparallel coiled coils (~50-nm) connect the hinge to ATPase head domains, which bind the N- and C-terminus of a kleisin subunit, RAD21, thereby forming a ring [47]. RAD21 can be bound by regulatory proteins such as HAWKs (HEAT repeat containing proteins Associated With Kleisins) including STAG1/STAG2 (SA1/SA2), PDS5A/PDS5B, and NIPBL [40,48–50]. For simplicity, herein we will refer to STAG1 or STAG2 as STAG and PDS5A or PDS5B as PDS5, though it is important to note that STAG1- and STAG2-cohesin play somewhat different roles [38,51–53]. The STAG protein is essentially always associated with cohesin unlike NIPBL and PDS5. NIPBL regulates cohesin loading on DNA [54–56] and is also required for loop extrusion *in vitro* [21,23]. PDS5, together with WAPL, regulates cohesin release from DNA [57–59]. Cohesin can switch between a single compartment state (S-K) and a two-compartment state (S and K) in an ATP-dependent manner (Figure 2a; see also [20,60–62] for a more comprehensive discussion). Despite great progress, many mechanistic and structural ambiguities remain and it is important to note that although cohesin-mediated DNA loop extrusion has now been observed *in vitro* [21–23], it remains to be demonstrated *in vivo*. Moreover, though models have been proposed [20,54,61,63,64], the molecular mechanism of cohesin extrusion remains unclear as does whether monomeric or dimeric cohesin extrudes loops [21,23,65]. Although cohesin can topologically enclose DNA inside its ring, loop extrusion seems to involve non- or pseudo-topological DNA engagement [21,23].

### CTCF

CTCF is an 11-Zinc Finger (ZF) DNA-Binding protein that is conserved across most animals, but absent from plants, *C. elegans* and yeast [66,67]. Mammalian CTCF has unstructured N- and C-terminal domains flanking the 11-ZF DNA-binding domain [68,69] (Figure 2b). Depending on the antibody used and the bioinformatic threshold, CTCF binds ~40,000–90,000 sites in mammalian genomes, of which ~30-60% are cell-type specific and with around half in intergenic regions and the other half at promoters, in introns or exons [39,66,70–72]. Consistent with CTCF regulating cohesin positioning on chromatin, but not cohesin loading onto chromatin, >90% of all cohesin ChIP-Seq peaks co-localize with CTCF [39,70,73–75], but CTCF depletion does not affect the amount of cohesin on chromatin, only its location [42,74,76].

### Loop extrusion

In the simplest formulation of the loop extrusion model, cohesin loads randomly on chromatin and begins extruding loops bidirectionally (Figure 2c). CTCF binds cognate sites and ‘passively’ waits for a cohesin complex to arrive. CTCF will block the extruding cohesin complex if, and only if, the CTCF site on DNA is in a convergent orientation such that cohesin first encounters its N-terminal domain (thus site ‘B’ is skipped in Figure 2c). We refer to cohesin’s preference for occupied CTCF binding sites in the convergent orientation as *the convergent rule* [11,18,31–34]. Since both CTCF and cohesin bind DNA dynamically [70], there is a significant probability that either CTCF or cohesin dissociates from DNA before they encounter each other. But if cohesin reaches two convergent and occupied CTCF binding sites, a CTCF loop is stabilized for an unknown duration and may appear as a ‘corner dot’ in a Hi-C map (Figure 1). Though the lifetime of such CTCF loops remain unknown, we have previously argued that they are likely dynamic [24,70]. Regardless, the loop may dissociate when CTCF or cohesin dissociates from DNA. If CTCF dissociates first, it is not known if cohesin remains or continues to extrude (Figure 2c). Polymer simulations of the simple loop extrusion model with just cohesin and CTCF generate contact maps similar to experimental maps at the level of TADs and loop domains [17,18,38,44,77]. This is a simplified picture of loop extrusion and it is important to note that loop extrusion inside the cell is likely not this simple: the transcriptional machinery, for example, is likely to also serve as a partial boundary to cohesin-mediated loop extrusion [76,78–80].

Key parameters for the simple loop extrusion model are: CTCF residence time, probability that a CTCF binding site is occupied, cohesin residence time, mean density of loop extruding cohesins on chromatin (upper bound estimated from G1 phase), and extrusion speed. Though the speed of extrusion in live cells remains unknown, various direct and indirect estimates have been reported: ~22.5 kb/min (cohesin in HCT116 cells [26]); ~6-12 kb/min (condensin II in chicken cells [81]); ~54 kb/min (bacterial SMC complex [82]), and ~30-60 kb/min on naked DNA *in vitro* [21,23]. The speed of cohesin extrusion is likely to differ between different local genomic regions and cell types. The other parameters have recently been estimated in mouse embryonic stem cells (mESC) [65] and human HeLa cells [83] (Figure 2d). One key insight is that CTCF forms a ‘permeable boundary’: all but the strongest CTCF binding sites are only occupied some of the time (on average, ~50% in mESC; ~68% in HeLa) [65,83]. Thus, an extruding cohesin can skip a convergent CTCF binding site a significant fraction of the time. This can explain why different loops may form at different cells, or in the same cell at different points in time [65,70,83], as well as the formation of nested loops and domains in Hi-C maps [30]. Whether cohesin exists and functions as a monomer, dimer, or oligomer remains debated [62,65,84,85] as does whether monomeric or dimeric cohesin extrudes loops [21,23]. Practically, this means that upper bound estimates of the density of loop extruding cohesin vary by a factor of two: ~2.67 to 5.34 per Mb in mESCs [65] and ~10-20 per Mb in HeLa [83] (Figure 2d). Thus, quantitative constraints on the loop extrusion model are beginning to emerge and obtaining such data for more cell types will further help reveal if and how loop extrusion parameters are regulated in a cell-type specific manner to regulate genome structure and function.

## One-step vs. multi-step CTCF-cohesin interactions to explain the convergent rule

**Figure 3.**
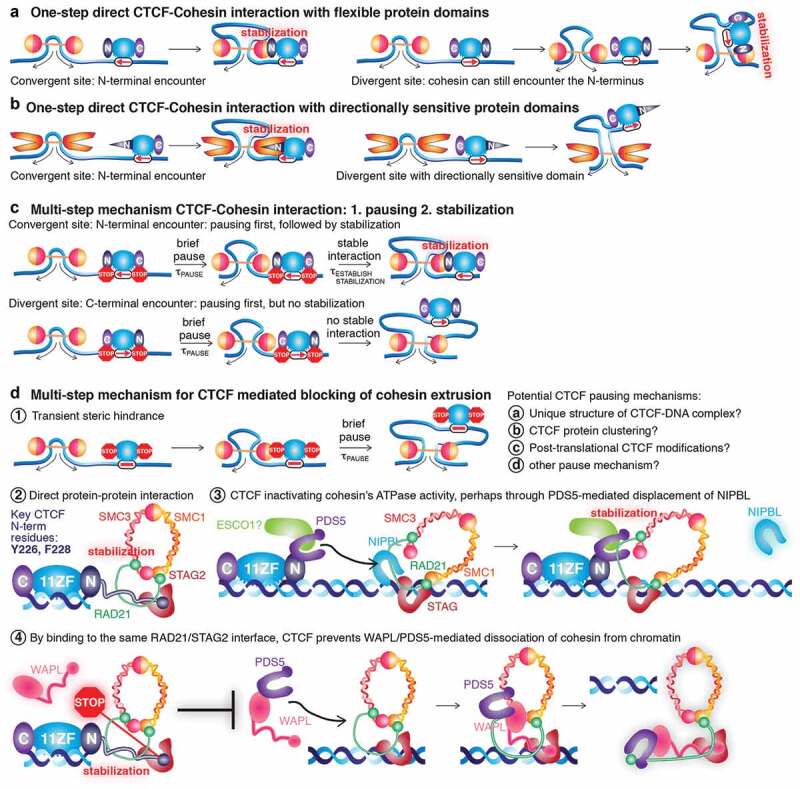
**One-step vs. Multi-step CTCF-cohesin interaction mechanisms**. (a) One-step CTCF-cohesin mechanism. If 1-step mechanism, it is not clear why cohesin could not extrude past the C-terminal domain of CTCF to interact with the N-terminal domain on the other side. (b) One-step CTCF-cohesin mechanism with directionally sensitive domains. For a one-step mechanism to work, the N-terminal CTCF domain and cohesin would both have to exhibit a directional sensitivity as illustrated. (c) Multi-step CTCF-cohesin mechanism. For an N-terminal encounter, pausing is eventually followed by stabilization. For a C-terminal encounter, pausing is not followed by stabilization, so cohesin eventually extrudes past or dissociates. (d) Instead of a one-step mechanism, a multi-step mechanism would involve transient pausing of cohesin next to CTCF (1), followed by stabilization of cohesin only from the N-terminal side of CTCF, through either direct protein-protein interaction (2), CTCF ‘turning OFF’ the cohesin motor (ATPase) perhaps mediated via PDS5A/B and/or ESCO1 (3), or through CTCF preventing WAPL-mediated release of cohesin from chromatin by CTCF binding to the same RAD21/STAG2 interface as WAPL does (4). It is important to note both that these mechanisms are not mutually exclusive, and that many other mechanisms could contribute.

The fact that cohesin would first encounter the N-terminus of CTCF when encountering a convergent CTCF site was originally used as a mechanistic explanation for the convergent rule. Given that cohesin extrudes loops very rapidly, this would require a near-instantaneous and near-deterministic interaction between cohesin and the CTCF N-terminus. However, if this interaction was a rapid and efficient ‘lock and key’ type interaction, it is not immediately clear why cohesin when encountering an incorrectly oriented CTCF binding site could not just extrude past the CTCF C-terminus to stably halt at the N-terminus (Figure 3a). Though it is conceivable that such a binding interface might be ‘directionally sensitive’ and only be properly presented from one orientation (Figure 3b).

Instead, recent studies [28,36–42] have proposed and substantiated a multi-step mechanism that can better explain the convergent rule involving pausing and stabilization [39–42] (Figure 3c). Along the lines of what is proposed in [42], cohesin extrusion is near-deterministically paused by CTCF, either dependent on or independent of CTCF binding site orientation. Cohesin pauses for a period, τ_PAUSE_, after which it will eventually extrude past CTCF. Next, assume that the N-terminus of CTCF can directly or indirectly stabilize cohesin, but that establishing stabilization is moderately slow though faster than the pause period (τ_ESTABLISH STABILIZATION_ < τ_PAUSE_) and also that the C-terminus cannot stabilize cohesin. Now if cohesin faces the C-terminal side of CTCF, cohesin will pause for duration, τ_PAUSE_, but since cohesin cannot be stabilized, cohesin will eventually extrude past or dissociate from DNA. Since cohesin extrudes quickly and since establishing stabilization is slow, it is extremely unlikely that stabilization will occur once cohesin extrudes over the N-terminus from a C-terminal encounter. In contrast, when cohesin encounters the N-terminal side first, it will pause and during the pause period τ_PAUSE_, it is very likely that stabilization is established before cohesin can escape since τ_ESTABLISH STABILIZATION_ < τ_PAUSE_. Once stabilized, a loop will be stabilized with duration τ_LOOP_.

Such a multi-step mechanism would be able to explain the convergent rule. Conceptually, this means the convergent rule can be divided into at least two components: 1) the mechanism of ***pausing*** and 2) the mechanism of ***stabilization***. Cohesin stabilization would likely be essential for forming sufficiently stable CTCF-loops to be visible as ‘corner peaks’ in Hi-C maps, whereas both pausing and stabilization would contribute to TAD insulation. Therefore, interfering with the stabilization mechanism would be expected to affect ‘loops’ more strongly in Hi-C maps than TADs, and this was indeed observed [40]. We therefore next consider potential mechanisms of pausing and stabilization.

### Mechanism of CTCF-mediated pausing of cohesin extrusion

At the most simplified level, given the size differences between CTCF (~3-5 nm) and cohesin (~50 nm), it is not immediately clear how CTCF could near-deterministically halt and pause an extruding cohesin (Figure 3d, 1). However, CTCF is an unusual DNA-binding protein and binds DNA for minutes [70,86,87] instead of seconds as seen for conventional transcription factors [88,89], and may have a unique ability to halt cohesin [90]. Here we consider a number of non-mutually exclusive possibilities.

First, CTCF is uniquely able to position ~20 nucleosomes around its DNA-binding sites [91–95], suggesting a mechanism whereby CTCF binding generates a unique chromatin microenvironment that could potentially serve as a steric hindrance pause signal. Consistently, CTCF has been reported to form a highly unusual DNA structure where it binds [96]. Thus, CTCF may simply pause cohesin through the unique 3D conformation of the CTCF-DNA complex [39], perhaps in a CTCF orientation independent manner. Though analyses of CTCF paralog CTCFL are informative here: CTCFL has a nearly identical 11ZF domain and motif preference to CTCF. While CTCFL cannot rescue CTCF-mediated genome organization [41], a CTCF-CTCFL chimera with the N-terminus and ZF1 + 2 from CTCF and ZF3-11 and C-terminus from CTCFL was able to largely rescue cohesin ChIP-Seq binding [39], though loop-resolution Hi-C analyses of this chimera were not performed. Beyond CTCF-DNA interactions, this roadblock model may also include CTCF-RNA interactions [36,37,39].

Second, we previously showed that CTCF forms clusters [70], whereby several to tens of CTCF proteins come together. Similarly, CTCF forms larger foci in senescent cells [97]. Recently, we showed that CTCF clustering is mediated partially through an internal RNA-Binding Region (RBR_i_) in CTCF, and consistent with CTCF clustering playing a role in genome organization, around one-third of all CTCF loops are lost in ΔRBR_i_-CTCF mESCs [36]. Mechanistically, a bulky cluster of CTCF may more efficiently pause an extruding cohesin – perhaps in a binding site orientation independent manner – which may explain why impaired CTCF clustering might also impair cohesin pausing, thereby leading to loop disruption. Thus, the current data is also consistent with CTCF clustering contributing to pausing cohesin.

Third, CTCF is subject to a number of post-translational modifications [66], some of which are quite large such as SUMOylation [98] and poly-ADP-ribosylation [99–101]. Early reports suggested that poly-ADP-ribosylation contributes to the insulation function of CTCF [99,100], and mutation of the 11 amino acids in the N-terminus reported to be poly-ADP-ribosylated as well as treatment with a PARP inhibitor, moderately decreased CTCF’s ability to stabilize cohesin at its binding sites as measured by ChIP-Seq [39]. These results are consistent with poly-ADP-ribosylation playing some role, albeit not an absolutely required one, in CTCF’s ability to pause cohesin extrusion.

It remains highly unclear exactly how CTCF might pause an extruding cohesin, and there may well be many other potential mechanisms such as DNA supercoiling and the chirality of the double helix [102]. It is also important to note that the three mechanisms discussed above – nucleosome positioning/chromatin structure, CTCF clustering, and poly-ADP-ribosylation – are not mutually exclusive but may instead be synergistic (Figure 3d, 1). As such, more work is urgently needed.

### Mechanism of CTCF-mediated stabilization of cohesin

Originally, a small region C-terminal to CTCF’s 11 Zinc Finger domain was reported to be necessary and sufficient for binding the STAG2 subunit of cohesin and this was assumed to be the only direct interaction between CTCF and the cohesin complex [103]. However, subsequent studies found that ΔRBR_i_-CTCF, whose deletion encompasses this region, co-immunoprecipitated cohesin equally well as wild-type CTCF [36,41,104], indicating that this region is not required for the biochemical CTCF-cohesin interaction, though it does contribute to loop formation [36]. Instead, despite different experimental systems and different read-outs, the recent papers all reached remarkable agreement that it is the CTCF-N-terminus as well as the first two Zinc Fingers that play key roles in the CTCF-cohesin interaction, though notably, neither region is autonomously sufficient [39–42,105]. Notably, placing the CTCF N-terminus on the C-terminal side does not fully recapitulate function [41,42]. Here, we discuss three mechanistic models, that are not mutually exclusive, for the interaction between CTCF and cohesin: direct protein-protein interaction, CTCF inactivating cohesin’s ATPase function, and CTCF stabilizing cohesin on DNA by antagonizing WAPL-mediated cohesin release (Figure 3d).

First, the simplest model invokes direct protein-protein interaction between CTCF and cohesin (Figure 3d, 2). This model is supported by the observed CTCF-cohesin co-immunoprecipitation [36,40,41,70,104,106] and Li *et al*. recently elucidated the structural basis for this interaction [40] by solving the crystal structure of a short CTCF peptide (N-terminal amino acids 222–231) bound to the RAD21-STAG2 interface. Thus, the CTCF N-terminus binds both RAD21 and STAG2, albeit relatively weakly (~0.6 μM) [40]. Thus, it is clear that a direct CTCF-cohesin protein-protein interaction is at play *in vitro* [40]. However, since it is relatively weak (~0.6 μM), it will be important to study in the future if there are additional interactions and how this interaction functions *in vivo*.

Second, CTCF could regulate cohesin’s ATPase activity directly or indirectly through cohesin regulatory proteins such as PDS5 or ESCO1. If CTCF could turn OFF ‘extrusion’, this might ‘lock’ cohesin in place and thereby stabilize a CTCF-cohesin loop (Figure 3d, 3). This model is suggested by studies by Wutz *et al*. [28,38]. WAPL binds PDS5 and releases cohesin from chromatin [58,59]. Depletion of WAPL greatly increases cohesin’s residence time on chromatin [58], leading to its reorganization into structures called ‘vermicelli’ and compaction of DNA [107]. Thus, in Hi-C maps, WAPL depleted cells exhibit many more loops or ‘corner peaks’ [28,108]. PDS5 depletion also increases cohesin’s residence time in G1 and leads to even more pronounced vermicelli than depleting WAPL alone [28]. Therefore, PDS5-depleted Hi-C maps ought to resemble WAPL-depleted Hi-C maps, but surprisingly, PDS5-depleted Hi-C maps show fewer loops, more closely resemble CTCF-depleted Hi-C maps, and strongly violate the convergent rule [28]. Specifically, if the CTCF binding site orientation played no role, 25% of CTCF loops would be expected to be convergent instead of the observed 65–92% [11,31,32,109]. But upon PDS5 depletion, only 30.9% of loops are bridged by convergent CTCF sites, implicating the PDS5 proteins in mediating the convergent rule [28]. Since NIPBL stimulates cohesin’s ATPase activity [110,111], is required for vermicelli formation [108], binds to the same cohesin interface as PDS5, and therefore competes with PDS5 for cohesin binding [49], Wutz *et al*. proposed that PDS5 could inactivate cohesin’s ATPase activity in a CTCF-dependent manner [28]. Consistently, NIPBL is required for cohesin extrusion *in vitro* [21,23] and is sub-stoichiometric compared to cohesin [111]. Thus, if following CTCF-mediated pausing of cohesin extrusion, the CTCF N-terminus and PDS5 could displace NIPBL or otherwise inactivate cohesin’s ATPase activity, this would mechanistically explain the convergent rule and stabilization of cohesin by CTCF. In this model, the role of PDS5 may depend on whether or not it partners with CTCF (turn off NIPBL) or WAPL (unload cohesin from DNA). For this model to explain bidirectional extrusion where cohesin can stop independently on the left and on the right, a dimeric form of cohesin associating with two NIPBL proteins which can be independently dissociated by CTCF/PDS5 would likely be required [21,62,65,85]. Finally, we note that direct support for PDS5’s ability to inhibit extrusion comes from *in vitro* single-molecule studies where WAPL-PDS5 were found to inhibit cohesin translocation on DNA [112].

**Figure 4.**
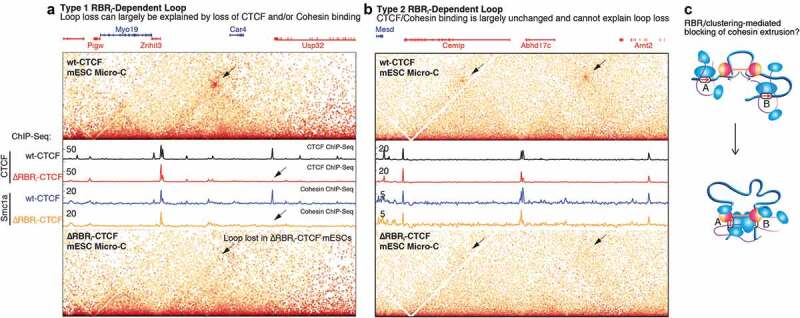
**Distinct classes of chromatin loops**. (a-b) Micro-C maps and CTCF and Cohesin (Smc1a) ChIP-Seq shown for wt-CTCF mESCs and ΔRBR_i_-CTCF mESCs, illustrating Type 1 RBR_i_-dependent loops that can be explained by loss of CTCF/cohesin binding (a) and Type 2 RBR_i_-dependent loops that cannot be explained by loss of CTCF/cohesin binding (b). (c) Sketch of a role for CTCF clustering in blocking cohesin extrusion. Figures 4a-c are partially reproduced and edited from [36] with permission.

Third, CTCF could stabilize cohesin on DNA by counteracting cohesin’s release from DNA by WAPL-PDS5 [58,59] (Figure 3d, 4). Cohesin exists in a dynamic equilibrium of binding and dissociation from chromatin and ~40% of cohesins are specifically associated with chromatin in G1 in mESCs [70]. This population may represent loop extruding cohesins. Intriguingly, Li *et al*. found that CTCF and WAPL bind the same RAD21-STAG2 interface of cohesin, with CTCF binding more strongly than WAPL [40]. Li *et al*. therefore proposed a model where CTCF stabilizes cohesin and loops by counteracting WAPL-mediating cohesin release from DNA. Consistently, CTCF depletion [38] and mutation of key RAD21-STAG2 interacting amino acids in CTCF (Y226A, F228A) [40] decreased cohesin’s residence time on DNA as measured by iFRAP. Relatedly, Wutz *et al*. recently found that STAG1-cohesin exhibits more SMC3 acetylation and a more stable residence time on DNA compared to STAG2-cohesin, which is less acetylated and binds DNA more dynamically [38]. Whereas ESCO2 acetylates cohesin only during S-Phase [113], ESCO1 acetylates cohesin throughout the cell cycle including during G1 [113], and ESCO1 is recruited to cohesin by PDS5 [114]. Thus, a related mechanism through which CTCF could stabilize cohesin would be CTCF-PDS5 mediated recruitment of ESCO1, which then acetylates and stabilizes cohesin at convergent CTCF sites [38]. Consistent with this model, RNAi-mediated depletion of ESCO1 and CTCF both decreased cohesin’s residence time [38] and SMC3 acetylation [38,76]. Consistent with CTCF and ESCO1 protecting cohesin from WAPL, co-depleting CTCF and WAPL results in the same cohesin residence time as WAPL depletion alone [38]. Taken together, the work of Li *et al*. and Wutz *et al*. suggest that CTCF stabilizes cohesin on DNA by counteracting WAPL-mediated release of cohesin from chromatin [38,40]. Potential mechanisms, which may be synergistic, include CTCF binding to the same RAD21-STAG2 interface as WAPL and thereby outcompeting WAPL [40] as well as CTCF-PDS5 mediated recruitment of ESCO1 to acetylate cohesin and thereby stabilize cohesin [38].

While recent work has greatly increased our knowledge of the direct and indirect ways CTCF and cohesin might form a stabilized loop maintenance complex and clarified the relevant domains and amino acids required, it is important to stress that we still do not understand the molecular mechanism. In this regard it is important to note that the three mechanisms discussed here – direct protein-protein interaction, CTCF inactivating cohesin’s ATPase activity and CTCF preventing WAPL-mediated release of cohesin from chromatin – are not mutually exclusive. Rather, they may be synergistic.

## Role of an internal RNA-binding region (RBR_i_) in CTCF in regulating CTCF clustering, target search, and chromatin looping

As an Extra View article to our recent two papers on the functions of an internal RNA-Binding Region (RBR_i_) in CTCF [36,43], we next discuss these and place them in the context of the proposed multi-step mechanism outlined above (Figure 3). Our studies were motivated by our previous observation that CTCF forms small clusters in mouse and human cells [70]. To understand the mechanism, we took a biochemical approach and generated an mESC line where the two endogenous alleles carry distinct epitope tags (3xFLAG-Halo-CTCF and V5-SNAP_f_-CTCF). FLAG- and V5-tagged CTCF co-IP’ed consistent with prior studies [99,104,115], but CTCF co-IP was insensitive to DNase but sensitive to RNase A treatment [36,104]. This suggests that CTCF self-associates in a manner that is directly or indirectly mediated by RNA [36,104]. Consistently, treatment with RNase reduces CTCF binding to chromatin [37,116]. The C-terminal Domain of CTCF was originally reported to be required for RNA binding and designated as CTCF’s RNA-Binding Region (RBR) [104,117] and deletion of amino acids 576–614 just C-terminal to the zinc finger domain was reported to drastically decrease RNA binding [104]. We refer to this internal 576–614 region as the RBR_i_ and it largely corresponds to mouse CTCF exon 10, which we homozygously deleted in an mESC line where CTCF has been homozygously Halo-tagged to generate Halo-ΔRBR_i_-CTCF (Halo-CTCF_Δ576-611_) [36]. Notably, although the RBR_i_ is physiologically important (the growth rate of ΔRBR_i_-CTCF mESCs is ~2-fold slower than wt-CTCF mESCs), ΔRBR_i_-CTCF only showed modestly reduced and not abolished RNA-binding *in vivo* and *in vitro* [36]. Consistently, subsequent studies found that ZF1 and ZF10 also contribute to CTCF RNA-Binding and likely bind RNA more strongly than the RBR_i_ [37,118]. It is important to note that whether CTCF-RNA interactions *in vivo* play more of a structural scaffold role or a direct interaction role remains unclear [119]. Regardless, using super-resolution PALM imaging we found that CTCF clustering is significantly reduced in ΔRBR_i_-CTCF mESCs, suggesting that CTCF clustering is partially mediated by the RBR_i_. Given the recent interest in Liquid-Liquid Phase Separation (LLPS) [120], it is worth noting that although CTCF exhibits many features associated with LLPS (Protein-RNA interactions, clustering, intrinsically disordered regions [68]), CTCF clustering is most likely not due to LLPS, at least not in mESCs and human U2OS cells. A key LLPS prediction is that protein over-expression above a critical concentration would cause all additional protein to enter the new phase [120–122]. However, even upon extremely high CTCF over-expression, CTCF remains relatively homogenously distributed in the nucleus [70], ruling out CTCF LLPS. Instead, it is worth noting that many TADs are demarcated by clustered CTCF binding sites [123].

In a parallel project, we had discovered that CTCF exhibits highly anomalous diffusion inside the nucleus [43]. CTCF exists in a DNA bound state (~49% bound to cognate sites and ~19% nonspecifically interacting with chromatin in mESCs) as well as a ‘freely’ diffusing state (~32%) [70]. We found that ‘freely diffusing’ CTCF exhibited anisotropic diffusion, such that following a step in one direction, CTCF is substantially more likely to return backwards. We developed a computational pipeline to analyze CTCF Single-Particle Tracking (SPT) data [43] and found that CTCF’s tendency to ‘return backwards’ primarily manifests itself at ~200 nm displacements. We explored a number of anomalous diffusion models [124,125], and found that the only model that can quantitatively explain our data is a model where CTCF gets transiently trapped in small ~200 nm zones inside the nucleus (typically for ms to tens of ms). We call this model Anisotropic Diffusion through transient Trapping in Zones (ADTZ) [43]. Our SPT data and theory suggested the existence of zones that trap CTCF and we therefore hypothesized that the zones would correspond to CTCF clusters. This is because clustering is due to (in)direct CTCF self-association, and self-association can also explain trapping. If this model is correct, 1) ΔRBR_i_-CTCF should exhibit strongly reduced anisotropic diffusion at ~200 nm scales and 2) wt-CTCF should exhibit anisotropic displacements predominantly in the vicinity of CTCF clusters. Both predictions were experimentally confirmed [43]. Thus, CTCF exhibits a novel mode of nuclear diffusion (ADTZ), which is likely mediated by CTCF clustering and the RBR_i_. Functionally, we found that the residence time for binding to cognate DNA sites is unaffected in ΔRBR_i_-CTCF, but that the specifically bound fraction is reduced. This means that it takes ΔRBR_i_-CTCF ~2.5-fold longer to find a cognate CTCF DNA-binding site compared to wt-CTCF [43]. Thus, CTCF exhibits a novel ‘facilitated’ or ‘guided’ nuclear target search mechanism where RBR_i_-mediated interactions accelerate the cognate DNA target search, without affecting the strength of cognate DNA binding itself [43].

Given the roles of the RBR_i_ in mediating CTCF clustering and target search and its physiological importance [36,43], we performed high-resolution Micro-C as well as ChIP-Seq and RNA-Seq to assess 3D genome organization in ΔRBR_i_-CTCF mESCs [36]. In the context of the multi-step CTCF-cohesin mechanism (Figure 3b), it is interesting that ΔRBR_i_-CTCF mESCs exhibit only moderate TAD and insulation defects, but that loops/corner peaks were strongly affected: of 14,372 loops, 57% (8,189 loops) and 39% (5,490 loops) were weakened by 1.5- and 2-fold, respectively. Thus, CTCF loops fall into at least two distinct categories, RBR_i_-independent and RBR_i_-dependent (Figure 4a-b). And we proposed a model where CTCF clustering might facilitate blocking or pausing of cohesin-mediated extrusion (Figure 4c).

Surprisingly, we found that RBR_i_-dependent loops can be further subdivided into two classes or types (Figure 4a,b). The loop in Figure 4a is an example of type 1 RBR_i_-dependent loops, anchored by two CTCF/Cohesin binding sites, at least one of which is lost upon RBR_i_ deletion. The left site is RBR_i_-independent: CTCF binds equally well with and without the RBR_i_ and cohesin is similarly recruited equally well with and without the RBR_i_. In contrast, CTCF and cohesin binding at the right-hand CTCF binding site is fully lost after RBR_i_-deletion in Figure 4a. The right site is thus RBR_i_-dependent. Since one CTCF-anchor is lost, this straightforwardly explains why the loop is RBR_i_-dependent and lost. Thus, type 1 losses are RBR_i_-dependent because at least one anchor is RBR_i_-dependent [36].

However, we were surprised to find a second category, type 2 RBR_i_-dependent loops (Figure 4b). Despite being bridged by CTCF/Cohesin anchors, these loops are lost without strong or obvious changes to CTCF/Cohesin binding, as measured by ChIP-Seq (Figure 4b) [36]. Notably, deletion of the ZF1 and ZF10 RBRs produced two similar types of loops: 1) loops lost that could be explained by loss of CTCF binding and 2) loops lost without obvious changes to CTCF binding as measured by ChIP-Seq [37]. Taken together, this suggests that while CTCF/Cohesin binding is *necessary* for loop formation, it is not *sufficient*. This means that ChIP-Seq is insufficient to read out the function of CTCF/cohesin mutants. Mechanistically, this suggests that CTCF and cohesin can form a ‘loop-incompetent’ complex, that is nevertheless sufficiently stabilized to generate a clear ChIP-Seq signal. This may explain why not all CTCF sites form loops and TADs. How ‘loop-competent’ and ‘loop-incompetent’ CTCF/Cohesin complexes differ mechanistically is an important question for future studies.

## Experimental considerations for studying CTCF-cohesin interactions

**Figure 5.**
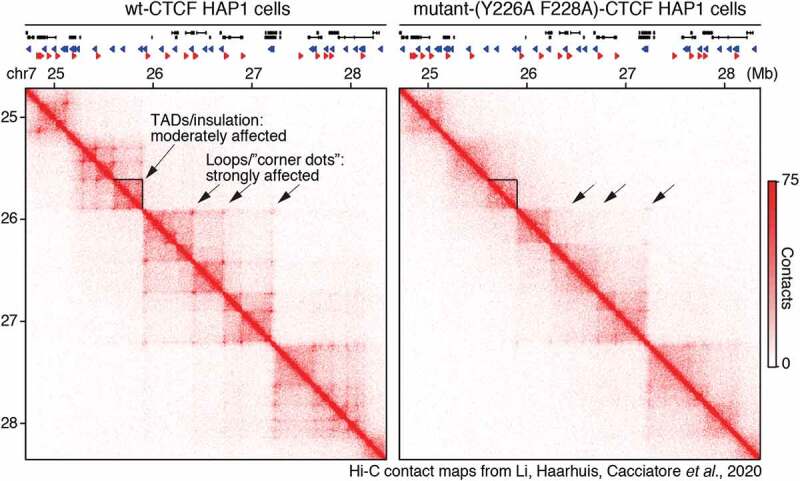
**CTCF-mediated loops can be disrupted with only modest effects on TADs/insulation**. (a-b) Hi-C contact matrices at 10 kb resolution of the HOXA locus in wt-CTCF and Y226A/F228A-CTCF HAP1 cells from [40].

Although recent papers have greatly increased our knowledge of how CTCF and cohesin interact, form chromatin loops, and insulate [28,36–42], the seemingly multi-step (Figure 3b-c) nature of the interaction has led to several surprising findings that should inform future studies and which we discuss here. Although CTCF/Cohesin DNA binding (typically measured with ChIP-Seq), TAD insulation and loop/corner peak formation are clearly related, it was found that they can be decoupled to a surprising extent [28,36–42]. First, loops/corner peaks can be disrupted without obvious changes to CTCF/cohesin binding as measured by ChIP-Seq [36,37] (Figure 4b). Second, loops/corner peaks can be near-completely disrupted with only moderate effects on TADs and insulation scores [36,37,40]. This point was clearly demonstrated by Li *et al*. in their Hi-C maps of mutant CTCF (Y226A, F228A HAP1 cells (Figure 5) [40]. Loops were almost entirely lost (from 2,756 loops to just 98 in mutant cells), yet, although TADs and insulation were clearly weakened, the effect was much more modest. Thus, loop-resolution Hi-C or Micro-C is necessary to distinguish effects on TADs and loops [36,37,40]. Similarly, since some CTCF mutants affect only a subset of loops [36,37], genome-wide analyses are necessary. In conclusion therefore, CTCF and Cohesin ChIP-Seq combined with loop-resolution Hi-C or Micro-C is required to fully ascertain the function of CTCF/Cohesin mutants, which unfortunately makes these experiments rather expensive.

Figures 5 is partially reproduced and edited from [40] with permission.

Moreover, since CTCF-null mice are embryonic lethal [126,127], studying CTCF mutants is experimentally challenging. One approach is generating endogenous mutants using genome-editing [36,40,43]. Advantages of this approach include: 1) it is clean – there is no endogenous protein left and 2) effects on physiology (e.g. growth rate, differentiation) are easily assessed; 3) gene expression is driven by endogenous regulatory elements. However, disadvantages include: 1) cannot study lethal mutations; 2) cannot distinguish primary from secondary effects; 3) has a low throughput. Thus, an alternative approach is to express wt- or mutant-CTCF in an inducible degron line (e.g. AID-tagged CTCF [28,29]) [37,41,42] or alternatively in a CTCF-mutant background [39] (though this is challenging to generalize and leads to co-expression of two versions of CTCF). The inducible complementation approach [37,41,42] is more general and 1) allows studying both lethal and non-lethal mutants; 2) allows some distinction between direct and secondary effects; 3) allows higher throughput. However, disadvantages include: 1) some AID-clones tend to have significant residual wt-protein; 2) it can difficult to achieve physiological expression. For the second point, it is important to consider both the mean and variance of the expression level distribution. E.g. in the inducible complementation system [41,42], the mean CTCF transgene expression was ~10-fold lower than endogenous CTCF level (~217,200 [65] vs. ~21,720 CTCF proteins per mESC). But the variance of transgene expression was also much greater (seemingly ~10-fold by FACS [41]). Greatly altered expression levels impairs our ability to distinguish effects of mutants from altered expression level. Therefore, since the inducible complementation approach is more generally applicable, an important future step will be to develop a next-generation version where the mean and variance of transgene expression matches those of endogenous CTCF. Ensuring endogenous expression levels will likely be especially important for cohesin, since overexpression of just one sub-unit causes the overexpressed subunit to primarily exist as monomers instead of being incorporated into cohesin complexes [70] and improper dosage of cohesin sub-units or regulatory proteins cause a range of diseases known as cohesinopathies [128].

## Conclusion and outlook

The 3D genome field is moving at an incredible pace and the last year alone has seen a number of important breakthroughs including: 1) the direct observation of cohesin extrusion *in vitro* for the first time [21–23], which strongly substantiates the loop extrusion model; 2) great new insights suggesting a multi-step CTCF-cohesin interaction mechanism to explain the convergent rule [28,36–42]; 3) the first structural insights into the CTCF-cohesin interaction [40]; 4) the development and application to mammalian cells of a chromosome conformation capture technique, Micro-C [36,129,130], which is capable of capturing enhancer-promoter interactions relevant to gene regulation that are otherwise largely invisible in Hi-C [131]. We end by highlighting a subset of the key challenges that lie ahead.

First, although the recent studies discussed here have shed great light on the interaction between CTCF and cohesin, suggested a multi-step mechanism for inhibition of loop extrusion, and revealed some of the necessary proteins and domains [28,36–42], we still do not understand the molecular mechanism. Furthermore, although referred to as the ‘convergent rule’, not all convergent CTCF sites form corner dots in Hi-C maps, and not all corner dots are bridged by convergent CTCF sites [11,31,32,109]. The development of *in vitro* [21,23] and cell-free extract [22] systems for loop extrusion now makes it possible to study the role of regulatory proteins (e.g. CTCF, WAPL, ESCO1, PDS5, nucleosomes, etc.) and mutants to probe the molecular mechanism of loop extrusion and its inhibition. Such approaches are likely to be particularly informative over the next few years.

Second, our mechanistic understanding is severely hampered by the current lack of structural insights. Full structural elucidation of full-length CTCF, cohesin and cohesin regulatory proteins at each step of the loop extrusion, pausing and stabilization process would yield profound insights.

Third, translation of these insights from *in vitro* to *in vivo* will be crucial, and the development and application of super-resolution live-cell imaging approaches will be necessary here.

Fourth, although TADs and loops were initially reported to be cell-type invariant [11,132], it has now become clear that cell differentiation is associated with widespread changes to TAD and loop organization [133–140]. Consistently, mutating RBRs in CTCF only disrupts a subset of loops [36,37] and CTCF binding can be regulated [141,142]. Understanding how and to which extent cell-type specific regulation of CTCF/cohesin-mediated chromatin looping takes place and is instructive for gene regulation is another important aspect to clarify in the future.

Fifth and along those lines, the roles of CTCF, cohesin and TADs in regulating gene expression remain controversial [143] and, although the experimental systems differ, the studies of CTCF mutants discussed here reported deregulation of several hundreds to a couple of thousand genes [36,37,39–41]. Distinguishing the roles of CTCF, cohesin and TADs in maintaining gene expression from establishing/inducing gene expression may be informative here [144,145].

Sixth, Micro-C has recently revealed the existence of ‘micro domains’ at the scale of kb to tens of kb, which are frequently anchored by proteins other than CTCF/cohesin including the transcriptional machinery [129,130]. Understanding the structure, regulation and function of these ‘micro domains’ will be important.

In summary, recent studies now suggest a multi-step mechanism for the interaction of CTCF and cohesin to explain aspects of the convergent rule [28,36–42], though we are likely still years away from understanding the molecular mechanisms. The 3D genome field shows no sign of slowing down and promises to be a particularly exciting field to continue to disentangle over the coming years.

## Extra view

Hansen AS, Hsieh THS, Cattoglio C, Pustova I, Saldana-Meyer R, Reinberg D, Darzacq X, Tjian R. Distinct Classes of Chromatin Loops Revealed by Deletion of an RNA-Binding Region in CTCF. *Molecular Cell*, 2019, doi:10.1016/j.molcel.2019.07.039

And also

Hansen AS; Amitai A; Cattoglio C; Tjian R; Darzacq X. Guided nuclear exploration increases CTCF target search efficiency. *Nature Chemical Biology*, 2019, doi:10.1038/s41589-019-0422-3
